# Commentary: Vitamin D and Pancreatic Cancer: A Pooled Analysis from the Pancreatic Cancer Case-Control Consortium

**DOI:** 10.3389/fonc.2015.00160

**Published:** 2015-08-06

**Authors:** Salvatore Chirumbolo

**Affiliations:** Department of Medicine, University of Verona, Verona, Italy

**Keywords:** cancer, pancreatic neoplasms, vitamin D, vitamin D deficiency, case reports

Waterhouse et al. criticized the association between vitamin D intake and the prevention of pancreatic cancer (1), an association that has been thoroughly reviewed in recent years (2, 3). Yet, randomized controlled clinical trials (RCTs) very rarely produced encouraging and reliable results on the field (4–7). Negative evidence in animal models and experimental studies (8, 9) should suggest that the chemopreventive role of 1,25(OH)_2_D_3_ deserve particular attention when dietary vitamin D_3_ is considered (10). Best correlations were reported on vitamin D_3_ deficiency and cancer malignancy (11–14) or on frequent dietary intake of vitamin D_3_ and tumor prevention (15). The chemopreventive role might closely depend on plasma bioavailability of 25(OH)D_3_ and genetic polymorphism of vitamin D receptor (VDR) (16, 17). Physicians are asking whether vitamin D_3_ supplementation may really contribute in preventing cancer (18, 19) and, at the same time, they suggested recommendations to fortify foods with supplemented vitamin D_3_, to achieve optimal levels of plasma 25(OH)D_3_ (20, 21).

Waterhouse et al. showed that cancer risk increased with higher levels of vitamin D intake, although they did not exclude the possibility that vitamin D obtained through ultraviolet exposure has a beneficial effect (1). In the future, 25(OH)D_3_ may become of major importance in assessing the role of the plasmatic content of vitamin D_3_ to prevent chronic diseases and cancer. Dietary vitamin D_3_ exhibited the same anti-cancer activity than 1,25(OH)_2_D_3_ in mice (9), a chemically modified form of 25(OH)vitD_3_ exerts a chemotherapeutic effect on neuroblastoma xenograft mouse model (22), an imbalance in plasma availability of 25(OH)D_3_ is considered a risk factor for carcinoma (23) and 25(OH)D_3_, likewise 1,25(OH)_2_D_3_, exerts an anti-inflammatory effect (24–26). Most of the recent evidence should suggest that plasma level of 25(OH)D_3_ has a fundamental role in warranting protection against chronic immune disorders and cancer (27). However, any approach to enhance 25(OH)D_3_ bioavailability with diet does not appear sufficient to improve vitamin D_3_-related outcome, due to genetic variability within the population (28). This evidence may appear therefore quite discouraging. Physicians are wondering how to focus onto vitamin D_3_ dietary intake to prevent chronic immune disorders and cancer. Yet, a proper determination of plasmatic 25(OH)D_3_ metabolites is highly recommended (29, 30). Clinical chemists have some difficulty in evaluating plasmatic 1,25(OH)_2_D_3_, particularly because it is rapidly degraded by 24-hydroxylases. Conversely, 25(OH)D_3_ biochemical activity should be attributed fundamentally to the 1-α-hydroxylated form, yet actually a more complex mechanism, involving multiple enzyme activity by P450 cytochromes and different metabolites, has been recently reviewed in Ref. (31). This should oblige nutritionists to be more cautious about the role of vitamin D_3_ supplementation in cancer prevention. Active vitamin D_3_ is a short-lived, potent hormonal molecule, whose efficacy seems to depend on the homeostatic level of circulating and available 25(OH)D_3_. The activity of the 25(OH)D_3_, is increased principally by the action of CYP27B1 but recent evidence has interestingly reported that a synthetic analog of 25(OH)D_3_, i.e., 5-hydroxy-16-ene-23-yne-D_3_, is neither modulated by CYP27B1 nor by CYP24A1 and expressed a potent anti-proliferative effect likewise 1,25(OH)_2_D_3_ (32). Furthermore, the use of synthetic analogs of 1,25(OH)_2_D_3_ appears quite promising in this field (33). Further, RCTs are needed to shed a light on the availability of newly introduced synthetic active forms of vitamin D_3_ for cancer prevention. The evidence should suggest that a possible way to enhance the anti-cancer activity of vitamin D_3_ is to increase 1,25(OH)_2_D_3_ effect by reducing the inhibitory action of CYP24A1, with molecules such as KD-35 or 4,5,6,7-tetrabromobenzimidazole (TBBz). This apparently simplistic point of view appeared quite encouraging (34, 35).

There are very few reports suggesting the possibility, through dietary intake, to improve the activity of 1,25(OH)_2_D_3_ as an immune cytokine and/or an hormone. CYP24A1 inhibitors, such as the isoflavone genistein, could be theoretically assumed with diet and they might potentiate the effect of 1,25(OH)_2_D_3_ in the immune response against cancer, although further randomized controlled trials are requested (36, 37).

Therefore, how to perform a correct dietary recommendation to promote vitamin D_3_ as a possible chemopreventive molecule? Western diet might induce or promote tumors, particularly when deficient or lacking vitamin D_3_ (38, 39). This should suggest why most of Western populations, living in industrialized countries, are often vitamin D_3_ deficient. In this perspective, the initial concern is to establish the proper dietary supplementation of vitamin D_3_, to achieve an optimal plasmatic level of 25(OH)D_3_. However, the correct supplementation of vitamin D_3_ should depend on sex and age, dietary habits, level of 25(OH)D_3_, geographical areas, individual’s gut microflora, and genetics of vitamin D_3_ metabolism (P450 cytochromes and VDR) (40–42) and this, at least theoretically, would oblige nutritionists, physicians, and caregivers to ask for a reappraisal of a Consensus Panel suggesting the proper vitamin D_3_ intake in relation to any of these factors (43). Due to the extreme difficulty in achieving this goal, any supplementation panel might be restricted to differential distributions in age clusters for both sexual groups and, anymore, to ensure an excess of circulating 25(OH)D_3_ in plasma. Notwithstanding, an excess of 25(OH)D_3_ may induce toxicity (44) and dampening the role of CYP24A1 in modulating 1,25(OH)_2_D_3_ activity may cause serious damage to kidney and calcium homeostasis (45). Therefore, as a severe plasma 25(OH)D_3_ deficiency is considered a bad prognostic marker for tumors (46), 25(OH)D_3_ plasma bioavailability should be considered a major bullet point in the nutritional research of chemopreventive molecules. Yet, researchers trust the fact that vitamin D_3_ should be particularly useful in cancer prevention (47). Plasma 25(OH)D_3_ might be considered of major important in the future, therefore, if associated with genomics and diet habits.

A suggested work flow to assess a possible correct intake of vitamin D_3_, as a supplementation factor in diet to prevent cancer, should henceforth consider also a genomic and nutrition screening, most probably according to steps described in Table 1.

**Table 1:** **Flow chart of vitamin D dietary evaluation**.

Pre-analytical stage	
a)	*The population undergoing vitamin D3 supplementation*: this point should be addressed by considering the main geographical area and whether population is coming from developing or industrialized countries (this fact should focus on the dietary habit), their sex, their age
b)	*Genetic polymorphism and mutational analysis*: particular genetic polymorphism for VDR should be highlighted (48–50). Moreover, genetic mutations for P450 cytochromes (particularly for CYP24A1) should be investigated (51, 52)
c)	*Metabolic homeostatic balance*: particular importance should be given to the metabolic homeostatic machinery held by the subject prior to his intake of vitamin D_3_ (calcidiol level, presence of insulin resistance or metabolic syndrome, metabolic markers, etc.)
d)	*Diet survey*: depending on the diet habit and life style, vitamin D_3_ supplementation might be accordingly adjusted, for a better performance

**Analytical stage**	

e)	*Data on vitamin D_3_ availability*: pharmacokinetics of vitamin D_3_, particularly when in association with chemopreventive drugs (53) should be known. A proper dosage of plasmatic calcidiol should be performed. A reappraisal on calcitriol determination should be conducted

**Post-analytical stage**	

f)	*Prospective studies and epidemiology*: further detailed studies on the association between vitamin D_3_ dietary intake and cancer development should give a sound contribution for the comprehension of the chemopreventive role of vitamin D_3_

## Conflict of Interest Statement

The author declares that the research was conducted in the absence of any commercial or financial relationships that could be construed as a potential conflict of interest.
